# Cannabinoid Signaling Recruits Astrocytes to Modulate Presynaptic Function in the Suprachiasmatic Nucleus

**DOI:** 10.1523/ENEURO.0081-19.2020

**Published:** 2020-02-03

**Authors:** Lauren M. Hablitz, Ali N. Gunesch, Olga Cravetchi, Michael Moldavan, Charles N. Allen

**Affiliations:** 1 Oregon Institute of Occupational Health Sciences, Oregon Health & Science University, Portland, OR 97239; 2 Department of Behavioral Neuroscience, Oregon Health and Science University, Portland, OR 97239

**Keywords:** astrocyte, circadian rhythm, endocannabinoid, GABA, suprachiasmatic nucleus

## Abstract

Circadian rhythms are 24-h cycles in physiology regulated by the suprachiasmatic nucleus (SCN) in the brain, where daily cues act on SCN neurons to alter clock timing. Cannabinoid signaling modulates SCN neuronal activity, although the mechanism remains unclear. We propose that neuronal activity generates endocannabinoid release, activating astrocyte Ca^2+^ signaling, which releases adenosine and activates adenosine-1 receptors (A1Rs) on the presynaptic axon terminals, decreasing GABA release. We demonstrated, in mice, that activation of cannabinoid-1 receptors (CB1R) with the agonist WIN 55,212-2 (WIN) reduced the miniature GABA receptor-mediated postsynaptic current (mGPSC) frequency by a mechanism that requires astrocytes and A1R. WIN activated an intracellular Ca^2+^ signaling pathway in astrocytes. Activating this intracellular Ca^2+^ pathway with designer receptors exclusively activated by designer drugs (DREADDs) also decreased the mGPSC frequency and required A1R activation. The frequency of spontaneous Ca^2+^ events, including those induced by depolarization of a postsynaptic SCN neuron, was reduced by blocking CB1R activation with AM251, demonstrating neuronal endocannabinoid signaling modulates astrocytic Ca^2+^ signaling in the SCN. Finally, daytime application of WIN or adenosine phase advanced the molecular circadian clock, indicating that this cannabinoid signaling pathway is vital for the timing of circadian rhythms.

## Visual Abstract

**Figure:**
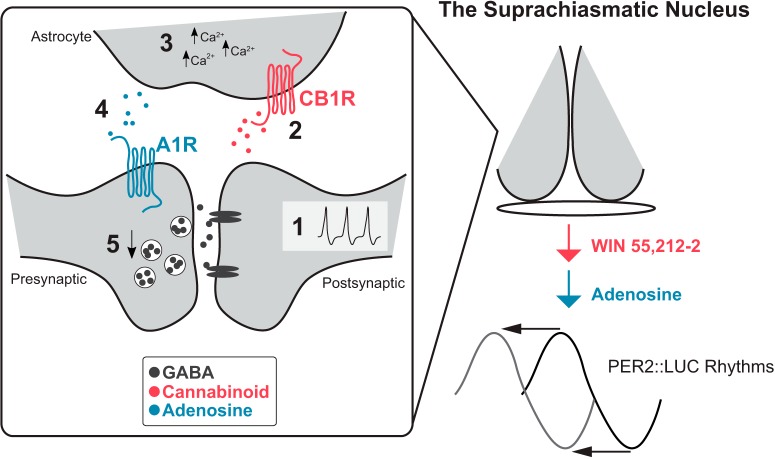

## Significance Statement

Astrocytes are a critical component of the neural network in the suprachiasmatic nucleus (SCN) required for the generation of precise circadian rhythms. We hypothesized that postsynaptic SCN neurons recruit astrocytes via endocannabinoid signaling to modulate presynaptic GABA release. We demonstrate, using a combination of approaches including whole-cell patch-clamp electrophysiology, GCaMP6 calcium imaging in astrocytes, pharmacology, and manipulation of intracellular signaling cascades using designer receptors exclusively activated by designer drug (DREADD) technology, that SCN neurons release cannabinoids that activate astrocyte intracellular Ca^2+^ signaling pathways to modulate GABA neurotransmission via adenosine. In addition to unraveling this novel signaling cascade in the SCN, we demonstrate that both CB1 receptor activation and adenosine phase advance the molecular circadian clock, suggesting an important role for astrocytes in modulating clock timing.

## Introduction

Circadian rhythms are 24-h cycles in behavioral and physiologic processes such as sleep/wake cycles, cognitive function, and hormone release. In mammals, the master circadian oscillator is located in the suprachiasmatic nucleus (SCN) where neurons expressing a molecular clock generate a 24-h timing signal. The network of SCN neurons and astrocytes, which also express molecular clocks, are required for a precise and stable circadian signal (Welsh et al., 2010; Jackson, 2011; Mohawk and Takahashi, 2011). A single astrocyte can interact with hundreds of synapses, enabling these cells to regulate network activity within brain regions (Pirttimaki and Parri, 2013). Astrocytes regulate clock gene expression and neuronal synchrony in culture systems, but how astrocytes regulate neuronal function within the SCN remains largely unknown (Beaulé et al., 2009; Marpegan et al., 2009, 2011; Barca-Mayo et al., 2017; Brancaccio et al., 2017; Tso et al., 2017; Svobodova et al., 2018). Astrocytes regulate neuronal function in multiple ways, from buffering extracellular ion and neurotransmitter concentrations (Bellot-Saez et al., 2017), to regulating blood oxygen and metabolism (Sonnay et al., 2018; Takata et al., 2018), and actively responding to external cues through intracellular signaling molecules such as Ca^2+^ and inducing the release of neuromodulators such as ATP, glutamate, or adenosine (Fiacco et al., 2009; Losi et al., 2014).

Endocannabinoids, endogenously generated lipophilic molecules, act as retrograde signals from neurons to regulate presynaptic neurotransmitter release via activation of G-protein-coupled cannabinoid-1 receptors (CB1Rs; Ohno-Shosaku et al., 2001; Wilson et al., 2001; Wilson and Nicoll, 2001; Araque et al., 2017). The production and metabolism of endocannabinoids have diurnal patterns, indicating they may be under circadian clock control (Valenti et al., 2004; Liedhegner et al., 2014; Koch et al., 2015). Cannabinoid receptor activation blocks light-induced phase shifts of circadian behavior (Sanford et al., 2008; Acuna-Goycolea et al., 2010). In addition, cannabinoid signaling increases neuronal firing within the SCN by decreasing presynaptic GABA release (Acuna-Goycolea et al., 2010). Given this evidence of interactions between the circadian and cannabinoid systems, surprisingly little is known about how cannabinoids alter SCN function and circadian clock timing. Endocannabinoids can alter neuronal function by activating astrocyte signaling pathways and the release of gliotransmitters (Navarrete and Araque, 2008). Here, we hypothesize that cannabinoid signaling activates an intracellular Ca^2+^ signaling pathway in astrocytes and releases neuromodulators to alter SCN neuronal function and ultimately change circadian clock timing.

## Materials and Methods

### Ethical approval

All animal care, handling, and housing were approved in advance by the Institutional Animal Care and Use Committee at Oregon Health & Science University.

### Animals and housing

Both male and female mice (three to six months of age) on a C57/BL6 background were used. Mice were group housed on a 12/12 h light/dark (LD) cycle with food and water ad libitum. GFAP-Cre mice (B6.Cg-Tg(Gfap-cre)73.12Mvs/J; The Jackson Laboratory, RRID:IMSR_JAX:012886), where Cre recombinase expression is driven by the promotor for the astrocytic marker glial fibrillary acidic protein (GFAP), were used to target expression to SCN astrocytes. Tail samples were sent to an external facility for genotyping (Transnetyx, Inc).

### Electrophysiology

Mice were terminally anesthetized between zeitgeber time (ZT)3 and ZT5 (ZT12 defined as lights off) with isoflurane followed by cervical dislocation and decapitation. All recordings were made between projected ZT7– ZT10. Brains were harvested, sectioned, and recorded using the patch clamp technique. Experimental treatments included WIN 55,212-2 (WIN; 3 μM; Sigma-Aldrich), AM251 (5 μM; Tocris), fluorocitrate (FC; 1 μM; Sigma-Aldrich), ACPT-II (200 μM; Tocris), CGS15943 (50 μM; Tocris), DPCPX (0.2 μM; Tocris), clozapine-N-oxide (CNO; 10 μM; Cayman Chemical), thaspsigargin (1 μM; Tocris), and adenosine (100 μM; Sigma-Aldrich). All miniature GABA(A) receptor-mediated postsynaptic currents (mGPSC; for rationale behind terminology, see Chavas and Marty, 2003; Woodin et al., 2003) recordings were performed in the presence of TTX (1 μM; Tocris) and CNQX (10 μM; Tocris) to block action potential driven synaptic transmission. During electrophysiological recordings, cells were voltage-clamped at –60 mV to ensure inward GABA currents. The microelectrode internal solution consisted of the following: 150 mM KCl, 20 mM HEPES, and 5 mM dextrose. Slicing solution consisted of the following: 111 mM NaCl, 6 mM Na(gluconate), 3 mM KCl, 1 mM Na_2_H_2_PO_4_, 4 mM MgCl_2_.6H_2_O, 26 mM NaHCO_3_, 11 mM dextrose, and 0.5 mM CaCl_2_.2H_2_O. Recording solution consisted of the following: 114 mM NaCl, 6 mM Na(gluconate), 2.7 mM KCl, 1 mM Na_2_H_2_PO_4_, 1 mM MgCl_2_.6H_2_O, 26 mM NaHCO_3_, 11 mM dextrose, and 2 mM CaCl_2_.2H_2_O. Baseline mGPSC frequency was defined as the average of three 20-s epochs before drug treatment. mGPSC analysis was performed using Igor Pro 7 (Wavemetrics) and data are presented as mean ± SEM. FC was applied to the slice 5 min before recording from a neuron, with a total treatment duration of ∼45 min. To reduce the possible physiologic damage to astrocytes produced by multiple FC exposures: after recording the response of a single neuron to FC application the FC was washed from the bath, the treated slice replaced with a naive slice, and a new experiment performed.

### Immunohistochemistry

Each mouse was deeply anesthetized with isoflurane and transcardially perfused with phosphate buffered saline 1× (PBS), pH 7.4, followed by 4% paraformaldehyde (PFA) in PBS. After perfusion, the brain was postfixed in PFA for 18 h at 4°C and cryoprotected by incubating overnight at 4°C first in 10% then 30% sucrose in PBS. Brain blocks were embedded in Shandon Cryochrome embedding medium (Thermo Fisher Scientific Inc.) and fast-frozen by dry ice mixed with 96% ethanol for 3–5 min. Coronal (20 μm thick) sections were cut with a Leica cryostat (CM1950, Leica Microsystems, Inc.), thaw-mounted onto pre-cleaned SuperFrost Plus glass slides, and dried at 37°C. Air-dried SCN-containing sections were hydrated in 0.1 M PB. To reduce background autofluorescence, the sections were incubated in an aldehyde-reducing agent 1% NaBH_4_ in 0.1 M PB for 30 min and rinsed copiously with multiple changes of 0.1 M PB until there were no signs of bubbles. The tissue was permeabilized with 0.3% Triton X-100 in TBS and non-specific binding was blocked by incubation in 5% normal donkey serum for 1 h at room temperature. For GFAP staining, the primary antibody was mouse anti–GFAP (1:1000, Millipore Bioscience Research Reagents MAB 3402, RRID:AB_94844). The secondary antibody was donkey anti-mouse Dy-Light 594 (1:1000, Jackson ImmunoResearch #715-585-151, RRID:AB_2340855). For neurofilament heavy chain (NFHC), primary antibody was chicken polyclonal anti-NFHC (1:500, Aves Labs, catalog #NFH, RRID:AB_2313552). The secondary antibody was donkey anti-chicken/CF 488A (1:500, Sigma SAB4600031, RRID:AB_2721061). For the neural/glial antigen-2 (NG2) staining, the primary antibody was mouse monoclonal anti-NG2 (1:250, Abcam catalog #ab50009, RRID:AB_881569). The secondary antibody was donkey anti-mouse Dy-Light 594 (1:1000, Jackson ImmunoResearch #715-585-151, RRID:AB_2340855).

### Stereotaxic surgery

Male and female GFAP-Cre mice were anesthetized with isoflurane and fixed in a stereotaxic frame. Next, two 90-nl boluses, 30 s apart of either AAV9.CAG.Flex.GCaMP6m.WPRE.SV40 (Penn Vector Core), AAV5-DIO-hM3Dq-mCherry (Addgene), or an equal mixture were bilaterally injected into the SCN to coordinates: *x*, –0.4, *y*, ±0.2, and *z*, –5.8 from bregma. SCN (150 μm) slices were prepared on days 10–21 after injection as described above. Successful injections were confirmed by observing expression of the green reporter GCaMP6 or mCherry expression from the Gq DREADD in the SCN.

### Calcium imaging

Ca^2+^ measurements were obtained by recording images at an excitation wavelength of 380 nm supplied via a Lambda 10-3 filter wheel (Sutter Instrument Company) passing through a UG11 optical filter to restrict harmonic wavelengths above 400 nm, reflected via a 400-nm DCLP dichroic mirror, and through a Leica 40×/0.80 UV water immersion objective (Leica Biosystems). Emitted light passed through a 510 ± 40 nm filter (Chroma Technology) and was recorded with a cooled charge-coupled device camera (CCD-1300-Y/HS, Princeton Scientific) with acquisition time and binning adjusted to minimize photobleaching and maximize recording speed via the digital imaging software Metafluor (Molecular Devices).

To record the long-term changes in [Ca^2+^]i due to bath application of WIN, images were sampled every 10 s. This was chosen based on previous work investigating Ca^2+^ signaling in cortical astrocytes, which demonstrated that most agonist-induced events lasted longer than 10 s (Poskanzer and Yuste, 2016). Regions of interest (ROIs) were selected based on visual identification of independent intensity differences from the rest of the slice. To evaluate changes of ROI intensity after drug treatment, the baseline was defined as the 300 s before test agent application. ΔF/F = (ROI intensity-baseline intensity)/baseline intensity. To eliminate experimenter bias in defining a positive or negative response, drug-induced change in Ca^2+^ was defined as a ΔF/F >2 SD away from baseline (Poskanzer and Yuste, 2016).

In separate experiments measuring faster, spontaneous Ca^2+^ events, and for paired electrophysiological recordings and GCaMP imaging of spontaneous Ca^2+^ events, images were acquired every 2 s, based on previous work in the SCN investigating spontaneous Ca^2+^ signals (Brancaccio et al., 2017), and to enable better resolution for washout experiments without photobleaching the epifluorescence. For these experiments, ΔF/F = ROI intensity/average slice intensity. Average slice intensity was used to eliminate large baseline shifts in intensity on drug treatment that may have been due to decreasing CB1R activity with AM251, or by photobleaching produced by the higher acquisition rate. An event was defined as a time point that was >2 SD away from the average intensity of the previous 30 s.

### Bioluminescence assays

Long-term organotypic slice cultures of the SCN were prepared from PER2::LUC mice. These slices allow for long-term recording of molecular clock rhythms that follow stable patterns of expression throughout the entire recording session, indicating that the SCN network remains largely intact (Yoo et al., 2004). Slices were treated within the first 4 d in the early subjective day, circadian time (CT)1–CT6 (where CT 12 is defined as peak bioluminescence), for 1 h with 3 μM WIN (Sigma-Aldrich), 0.09% DMSO, 0.2 μM DPCPX, a combination treatment of WIN and DPCPX, 100 μM adenosine (Sigma-Aldrich), or 0.38% sterile water. Data were acquired and analyzed with Lumicycle Analysis software (Actimetrics, Inc). Baseline drift in Lumicycle recordings was corrected by subtracting a third order or less polynomial followed by fitting with a damped sine wave. Only recordings with >75% goodness-of-fit to a dampened sine wave were used for analysis. Phase shifts were calculated by comparing two predictions for the time of the first peak post-treatment: one prediction calculated from at least three cycles before treatment and a second prediction derived from at least three cycles after treatment. The difference between these two predictions indicated the size of the phase shift (Besing et al., 2012; Hablitz et al., 2014).

### Statistical analysis

All statistical analysis was performed with PASW Statistics 18 (IBM Analytics). For comparisons of means in samples with normal distributions and homogeneous variances (as indicated by a Levene’s test), an independent samples *t* test or ANOVA was used for comparisons between two means or two or more means, respectively, followed by Fisher’s Bonferroni adjusted *post hoc* test when necessary. In cases of a non-normal distribution (as indicated by a Shapiro–Wilk test) or unequal variances (Levene’s test), a nonparametric Mann–Whitney *U* test or Kruskal–Wallis test was used for comparisons between two means or two or more means, respectively, followed by a median test for *post hoc* analyses. Significance was ascribed at *p* < 0.05. A repeated measures analysis was used for change over time within a cell or a slice, when appropriate. In the case of a non-normal distribution, a Friedman test followed by *post hoc* Wilcoxon signed-rank tests with the alpha level Bonferroni adjusted for multiple comparisons was used.

## Results

### WIN causes astrocyte-dependent changes in mGPSC frequency

**Figure 1.**
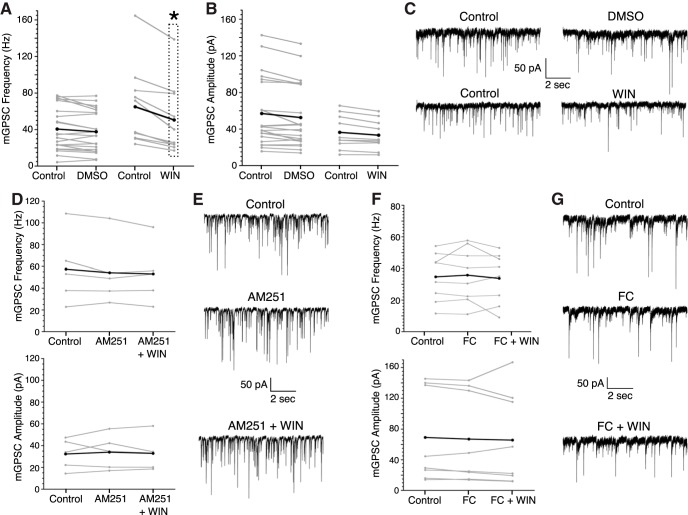
WIN decreases mGPSC frequency in a CB1R and astrocyte-dependent manner. For all frequency and amplitude plots gray lines are individual cells, black lines indicate the group average. ***A***, Individual cell and mean mGPSC frequencies before and after treatment with either DMSO (0.01%) or WIN (3 μM). ***B***, Individual cell and mean amplitudes of mGPSCs before and after DMSO or WIN application. ***C***, Representative mGPSC current recordings before and after treatment with DMSO or WIN. ***D***, Individual cell and mean mGPSC frequency (top) and amplitude (bottom) pretreatment, with AM251 (5 μM), and with both AM251 (5 μM) and WIN (3 μM). ***E***, Representative mGPSC recordings from a single neuron before and during treatment, with AM251, and with AM251 and WIN. ***F***, Individual cell mGPSC frequency (top) and amplitude (bottom) before and during application of FC (1 μM), and with both FC (1 μM) and WIN (3 μM). ***G***, Representative mGPSC recordings from a single neuron before and during treatment with FC, and with FC and WIN; *p < 0.001, repeated measures analysis.

The CB1/2R agonist WIN was used to examine the effect of CB1/2R activation on mGPSC frequency in the SCN (Fig. 1). Bath application of WIN (3 μM) significantly decreased the mGPSC frequency but not amplitude compared to DMSO (0.01%) controls (WIN: –25.6 ± 3.6% vs DMSO: –5.2 ± 4.3%, Fig. 1*A–C*; for frequency and amplitude averages and normalized averages, see Tables 1, 2 for statistical test results, see Table 3). The CB1R antagonist, AM251 (5 μM) completely blocked the WIN-induced reduction of the mGPSC frequency (AM251+WIN: –5.3 ± 3.1%). AM251 alone did not significantly change the mGPSC frequency or amplitude compared to 0.01% DMSO controls (Fig. 1*D*,*E*; Tables 1-Tables 3). These data are consistent with previous work demonstrating CB1R mediated inhibition of GABA release from presynaptic terminals (Acuna-Goycolea et al., 2010).

**Table 1.** mGPSC frequency data (Hz) for whole-cell electrophysiology experiments

Frequency (Hz)										
			Group means				Mean of within cell ratio			
Treatment 1	Control		Treatment 1		Treatment 2		Treatment 1/control		Treatment 2/control	
Mean	SEM	Mean	SEM	Mean	SEM	Mean	SEM	Mean	SEM	
DMSO	40.57	5.22	37.64	4.87	-	-	0.95	0.04	-	-
WIN	64.98	13.77	50.50	12.36	-	-	0.74	0.04	-	-
CNO	48.56	11.04	41.07	10.01	-	-	0.82	0.04	-	-
Adenosine	42.03	4.63	33.75	3.80	-	-	0.79	0.05	-	-
Treatment 2: WIN (3 μM)										
AM251	57.46	14.60	54.22	13.32	53.14	12.32	0.97	0.06	0.95	0.03
FC	34.70	5.46	35.78	6.13	33.73	5.67	1.02	0.04	0.98	0.09
ACPTII	34.14	5.07	32.86	5.08	28.30	4.65	0.96	0.03	0.81	0.04
CGS	38.78	8.25	38.50	6.93	36.86	6.53	1.04	0.05	1.00	0.05
DPCPX	44.92	4.83	42.29	4.77	42.48	5.40	0.94	0.03	0.99	0.06
Treatment 2: CNO (10 μM)										
DPCPX	39.08	3.25	37.87	3.79	34.83	3.01	0.96	0.04	0.94	0.07

The treatment/control ratio was calculated for each recorded neuron and then averaged for each treatment group.

**Table 2.** mGPSC amplitude data (pA) for the whole-cell electrophysiology experiments

Amplitude (pA)										
	Group means						Mean of within cell ratio			
Treatment 1	Control		Treatment 1		Treatment 2		Treatment 1/control		Treatment 2/control	
Mean	SEM	Mean	SEM	Mean	SEM	Mean	SEM	Mean	SEM	
DMSO	45.75	8.88	43.41	8.01	-	-	0.92	0.02	-	-
WIN	36.76	6.55	34.06	5.70	-	-	0.93	0.02	-	-
CNO	39.88	8.40	39.20	7.53	-	-	1.03	0.07	-	-
Adenosine	38.54	4.72	37.43	4.56	-	-	0.97	0.03	-	-
Treatment 2: WIN (3 μM)										
AM251	34.85	7.38	37.40	8.03	32.90	7.08	1.06	0.09	1.04	0.10
FC	72.48	27.41	70.96	25.97	65.65	21.34	0.96	0.03	0.91	0.07
ACPTII	45.31	7.99	43.76	7.02	41.32	6.20	0.99	0.03	0.96	0.05
CGS	48.69	10.49	48.96	10.34	46.40	10.30	1.01	0.02	0.94	0.03
DPCPX	61.72	14.98	57.29	13.14	55.12	11.72	0.96	0.03	0.92	0.04
Treatment 2: CNO (10 μM)										
DPCPX	41.30	5.74	40.68	6.48	37.66	6.37	0.96	0.03	0.87	0.05

The treatment/control ratio was calculated for each recorded neuron and then averaged for each treatment group.

**Table 3.** Statistics for the data shown in Figures 1-Figures 3

Figure	Statistical test	Statistics output	*N*
Fig. 1*A*,*B*			WIN: 10 neurons, 4 mice, DMSO: 21 neurons, 7 mice
mGPSC frequency: DMSO × WIN	ANOVA	*F*_(1,28)_ = 14.704, *p* = 0.001	
mGPSC amplitude: DMSO × WIN	ANOVA	*F*_(1,29)_ = 0.598, *p* = 0.445	
Fig. 1*D*			AM251: 5 neurons, 3 mice
mGPSC frequency: DMSO × AM251	ANOVA	*F*_(1,30)_ = 1.153, *p* = 0.292	
mGPSC amplitude: DMSO × AM251	ANOVA	*F*_(1,30)_ = 2.975, *p* = 0.095	
mGPSC frequency: AM251 × AM251 + WIN	Repeated measures ANOVA	*F*_(2,8)_ = 1.719, *p* = 0.239	
Fig. 1*F*			FC: 8 neurons, 3 mice
mGPSC frequency: DMSO × FC	ANOVA	*F*_(1,28)_ = 3.52, *p* = 0.071	
Fig. 3*A*,*B*,*E*,*F*			WIN: 4 mice, 6 slices, 38 soma (s), 194 non-soma (ns)
Increase magnitude: treatment (WIN/DMSO) × ROI (s/ns)	Kruskal–Wallis	H(3) = 169.052, *p* < 0.001	DMSO: 3 mice, 4 slices, 70 s, 86 ns
Increase magnitude DMSO: s × ns	Median *post hoc*	*p* = 1	
Increase magnitude WIN: s × ns	Median *post hoc*	*p* = 1	
Increase magnitude (both s and ns): DMSO × WIN	Median *post hoc*	*p* < 0.001	
Decrease magnitude: treatment (WIN/DMSO) × ROI (s/ns)	Kruskal–Wallis	H(3) = 60.729, *p* < 0.001	
Decrease magnitude DMSO: s × ns	Median *post hoc*	*p* = 1	
Decrease magnitude WIN: s × ns	Median *post hoc*	*p* = 1	
Increase magnitude (both s and ns): DMSO × WIN	Median *post hoc*	*p* < 0.01	
Fig. 3*C*,*G*			TTX + CNQX + WIN: 4 mice, 5 slices, 19 s, 93 ns
Increase magnitude: treatment (WIIN/ TTX + CNQX + WIN) × ROI (s/ns)	Kruskal–Wallis	H(3) = 13.35, *p* = 0.004	
Increase magnitude ns: WIN × TTX + CNQX + WIN	Median *post hoc*	*p* = 0.017	
Decrease magnitude: treatment (WIIN/ TTX + CNQX + WIN) × ROI (s/ns)	Kruskal–Wallis	H(3) = 2.213, *p* = 0.529	

After demonstrating that WIN decreases the frequency of mGPSCs, we next sought to determine whether astrocytes played a role in cannabinoid signaling. Astrocytic metabolic function was inhibited with FC (1 μM), an inhibitor of the Krebs cycle preferentially taken up by astrocytes (Navarrete and Araque, 2008). FC application did not significantly change mGPSC frequency or amplitude compared to 0.01% DMSO controls (Fig. 1*F*,*G*). However, FC completely occluded the WIN reduction of the mGPSC frequency (FC+WIN: –2.0 ± 8.58%), and had no effect on the mGPSC amplitude, indicating that proper astrocytic functioning was necessary for the presynaptic effects of WIN (Fig. 1*F*,*G*; Tables 1-Tables 3).

### The CB1/2R agonist WIN induces an increase in intracellular astrocytic Ca^2+^

**Figure 2.**
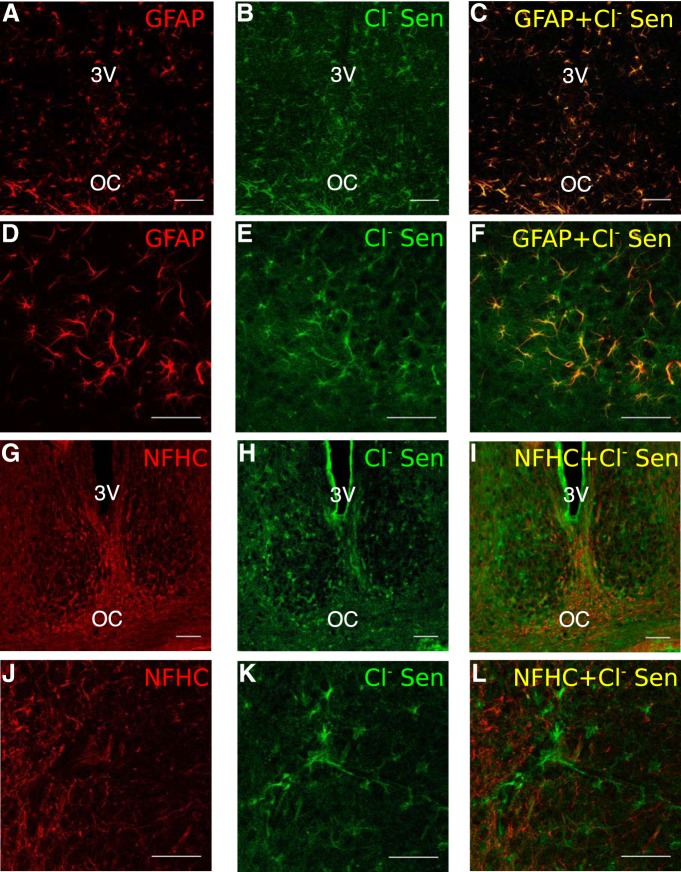
Chloride sensor expression in GFAP-Cre+ animals is specific to GFAP expressing astrocytes and not neurons or glial precursor cells. ***A–F***, GFAP labeling (left), Cl sensor YFP fluorescence (center), and colocalization (right) within the SCN of GFAP-Cre+/Cl sensor+ animals. ***G–L***, NFHC neuronal marker (left), Cl sensor YFP (center), and lack of colocalization (right) within the SCN. Scale bars are all 50 μm, images ***A–C*** and ***G–I*** were taken at 20× and images ***D–F*** and ***J–L*** were taken at 40×.

To understand how cannabinoids influence astrocyte signaling, we targeted astrocytes by using a mouse line where Cre recombinase expression is driven by the astrocytic marker GFAP. To validate the specificity of the Cre recombinase expression to astrocytes in the SCN, we bred GFAP-Cre mice with the chloride sensor (Cl^-^ sensor) mouse line, a chloride reporter that has robust expression and a strong fluorescent signal (Waseem et al., 2010; Klett and Allen, 2017). We then compared the expression of the Cl^-^ sensor to that of neuronal and glial cells markers in the SCN. The GFAP staining and fluorescent Cl^-^ sensor signal co-localized completely (Fig. 2*A–F*). Cl^-^ sensor expression was not detected in cells positive for NFHC (Fig. 2*G–L*) or the Neural/Glial antigen proteoglycan (data not shown), a marker for polydenrocyte glia (Nishiyama et al., 2009). Note that NFHC was used instead of more commonly used neuronal markers, such as neuronal nuclear antigen and neuron specific enolase, which are barely detectable in SCN neurons (Morin et al., 2011; Moldavan et al., 2015). These data demonstrate that the GFAP-Cre mice are an appropriate model to target astrocytes in the SCN.

**Figure 3.**
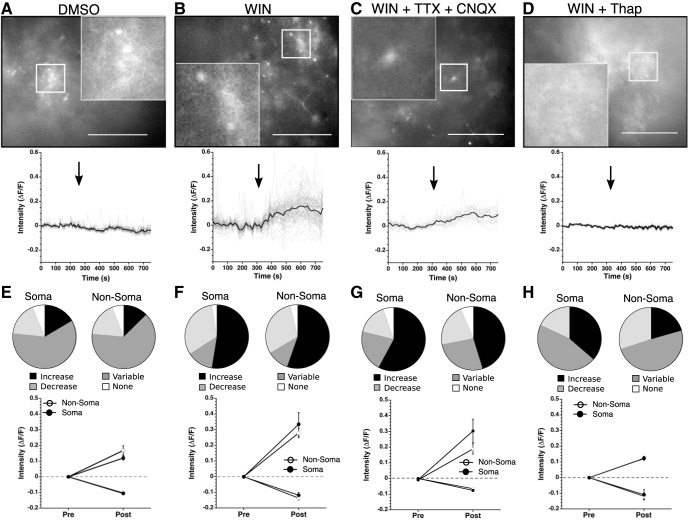
WIN activates an astrocytic Ca^2+^ signaling pathway. ***A–D***, top, Representative GCaMP6 images from slices treated with DMSO (0.01%), WIN (3 μM), WIN (3 μM) + TTX (1 μM) + CNQX (10 μM), and WIN (3 μM) + thapsigargin (1 μM; Thap). White scale bar is 50 μm, images taken at 40× and the inserts magnified 120×. Bottom, Representative traces showing the change in fluorescence from all regions measured (gray) and average response (black) from the slice depicted above. Black arrow indicates beginning of treatment. ***E*–*H***, top, Pie charts depicting percentage of soma and non-soma regions across all slices responding with an increase (black), decrease (dark gray), variable response (light gray), or no response (white) to DMSO, WIN, WIN + TTX + CNQX, or WIN + Thap (left to right). Bottom, Cumulative average fluorescence increase during baseline and drug treatment for soma and non-soma regions across all slices.

Endocannabinoids recruit astrocytes to mediate synaptic transmission by initiating intracellular Ca^2+^ signaling cascades (Navarrete and Araque, 2010; Bindocci et al., 2017). Here, we tested the hypothesis that activation of CB1/2Rs activates an intracellular Ca^2+^ signaling pathway in SCN astrocytes (Fig. 3). An adeno-associated virus containing GCaMP6, an intensity based Ca^2+^ reporter that is flanked by loxP sites (Chen et al., 2013), was injected into the SCN of GFAP-Cre+ animals to enable monitoring of Ca^2+^ signaling in SCN astrocytes. Astrocyte regions were defined as soma or non-soma by shape; somas were identified as more circular with thin processes branching from the center. This distinction was made because astrocytes differentially, spatiotemporally, regulate Ca^2+^ influxes throughout their somas and processes (Shigetomi et al., 2013; Tong et al., 2013; Bindocci et al., 2017). Increases or decreases of intracellular Ca^2+^ were defined as events if the amplitude was >2 SD from baseline, with variable responses showing both a significant increase and a significant decrease (Irwin and Allen, 2013). WIN (3 μM) application increased [Ca^2+^]i in 52.5% of the somas. The non-soma regions showed similar responses with increased [Ca^2+^]i in 55.2% (Fig. 3*B*,*F*). Controls were treated with 0.01% DMSO, the concentration present in the WIN solution. In contrast to the large percentage of cells in which WIN increased [Ca^2+^]i, DMSO increased [Ca^2+^]i in only 16.4% of soma and 12.6% of non-soma regions (Fig. 3*A*,*E*). The average increase in response to WIN application was 0.287 ± 0.028 ΔF/F, which was significantly higher than the DMSO average increase of 0.143 ± 0.016 ΔF/F (Table 3). WIN application produced small but significant decreases in [Ca^2+^]i (WIN: –0.134 ± 0.010 ΔF/F vs DMSO: –0.105 ± 0.003 ΔF/F). There was no significant difference between the soma and non-soma in the magnitude of the [Ca^2+^]i increases or decreases under WIN or DMSO. Those ROI that had variable responses, both exceeding the threshold for increased Ca^2+^ and decreased Ca^2+^ during the recording period, had low magnitude responses similar to DMSO indicating these responses may be normal, WIN-independent fluctuations of Ca^2+^ within the slice (Fig. 3*E*,*F*). These data indicate that WIN increases astrocytic [Ca^2+^]i, with low magnitude decreases or variable responses resembling vehicle control Ca^2+^ dynamics.

To determine whether this increase of astrocyte [Ca^2+^]i was driven by changes in neuronal activity, we performed the same experiment in the presence of TTX and CNQX to block synaptic transmission (Fig. 3*C*,*G*). Similar to the previous experiment, WIN application increased [Ca^2+^]i in 57.9% of the soma regions and 45.2% of the non-soma regions, indicating action potential firing by SCN neurons was not necessary for WIN to activate astrocyte [Ca^2+^]i pathways. Additionally, we found increased non-soma [Ca^2+^]i response in the presence of TTX and CNQX compared to the non-soma response to WIN (non-soma WIN: 0.279 ± 0.030 ΔF/F, non-soma WIN + TTX/CNQX: 0.198 ± 0.036 ΔF/F; Table 3). There were no significant differences in the soma responses to WIN. This indicates that inhibition of neuronal firing may alter a neuronal signal or signals, such as endocannabinoids, that regulate the [Ca^2+^]i in non-soma regions of SCN astrocytes (Metna-Laurent and Marsicano, 2015). Of the cells that showed a decreased [Ca^2+^]i, there were no significant differences in the amplitude of the Ca^2+^ responses between soma and non-soma regions, regardless of the presence of TTX+CNQX. In summary, WIN increases [Ca^2+^]i independent of neuronal firing, but neuronal signaling may modulate the magnitude of non-somatic Ca^2+^ responses.

To test the hypothesis that WIN-induced [Ca^2+^]i increases are dependent on intracellular Ca^2+^ stores, we used thapsigargin, a non-competitive inhibitor of the sarco/endoplasmic reticulum Ca^2+^ ATPase, to deplete intracellular Ca^2+^ stores (Fig. 3*D*,*H*). Thapsigargin (1 μM) increased [Ca^2+^]i by 380.4 ± 165.5% from pre-treatment levels, with Ca^2+^ levels dropping off significantly after the maximum as internal stores were depleted. WIN (3 μM) was applied after the [Ca^2+^]i reached a steady state value for at least 300 s. Because thapsigargin depletes intracellular Ca^2+^ stores, and the intensity of the GCaMP6 signal is dependent on [Ca^2+^]i, fewer somas were identifiable after Ca^2+^ depletion (before thapsigargin treatment: 35 visible somas, after: 11 visible somas). Thapsigargin treatment reduced the number and magnitude of WIN-induced [Ca^2+^]i increases indicating that WIN is activating Ca^2+^ release from intracellular stores. Of the 11 soma regions, only 4 showed an increase. Similarly, only 20 of the 97 non-soma regions showed an increase (Fig. 3*H*). The magnitude of these responses were smaller than responses without thapsigargin, although only the non-soma group, where the sample size was larger, was statistically significant (non-soma: 0.122 ± 0.011 ΔF/F, soma: 0.123 ± 0.012 ΔF/F; Table 3). These data demonstrate that activation of CB1 receptors by WIN activates an intracellular Ca^2+^ signaling pathway in SCN astrocytes.

### Neurons utilize endocannabinoid signaling to alter astrocyte function

**Figure 4.**
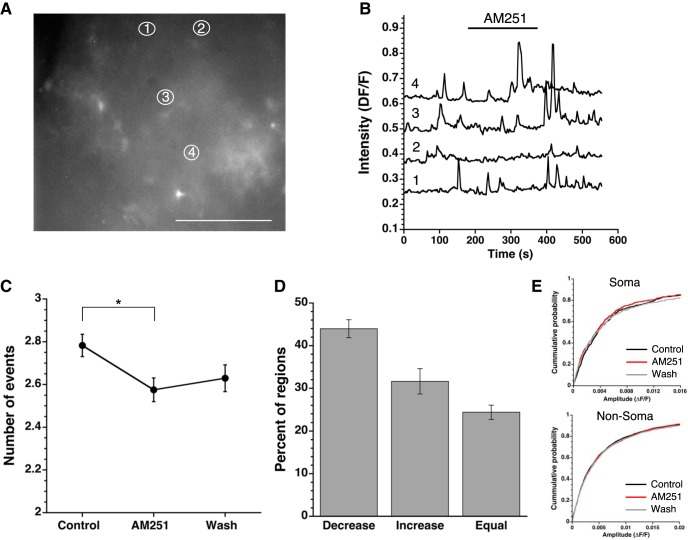
Blockade of CB1R alters the frequency of spontaneous astrocytic Ca^2+^ events. ***A***, Representative GCaMP6 image. White scale bar is 50 μm, image taken at 40×. White, numbered ellipses are the ROIs plotted in ***B***. ***B***, Representative traces of intensity 180 s before, during, and after AM251 treatment (indicated by black bar). Numbers 1–4 correspond to numbered regions in ***A***. Note that the event examples were picked to reflect the varying responses to AM251 with traces 2 and 3 showing a clear reduction in frequency. ***C***, Average event numbers across all experiments of regions before, during, and after AM251 treatment depicted as mean ± SEM (*p < 0.05, Friedman test). ***D***, Average percentage of regions responding with a decrease, increase, or no change on treatment with AM251 across all experiments (mean ± SEM). ***E***, Cumulative probability curves of amplitude for both soma (top) and non-soma (bottom) regions before, during, and after treatment with AM251.

We demonstrated that WIN-induced CB1R activation increased astrocytic [Ca^2+^]i (Fig. 3). However, agonist-induced activation of CB1R does not address endogenous, spontaneous Ca^2+^ signaling in astrocytes. Spontaneous Ca^2+^ events change both local and network wide intracellular signaling within and across astrocytes, disruption of which has been linked to neuropathology (Shigetomi et al., 2016). We determined the role of endogenous cannabinoid signaling in regulating these spontaneous Ca^2+^ events within GCaMP6 expressing astrocytes by applying AM251 (Fig. 4). Total event numbers were summed for the 3 min prior, 3 min during, and 3 min after AM251 treatment. AM251, reduced the frequency of [Ca^2+^]i events in 42.9% of somas. In non-soma regions AM251 reduced the number of spontaneous Ca^2+^ events in 44.1% of regions (Fig. 4*D*). We found no difference in event amplitude between soma and non-soma regions before, during, or after AM251 treatment (Fig. 4*E*; Table 4).

**Table 4.** Statistics for the data shown in Figure 4

Figure	Statistical test	Statistics output	*N*
Fig. 3*D*,*H*			Thapsigargin + WIN: 3 mice, 4 slices, 11 soma (s), 97 non-soma (ns)
Increase magnitude: treatment (WIN/ Thap + WIN) × ROI (s/ns)	Kruskal–Wallis	H(3) = 14.685, *p* = 0.002	
Fig. 4			AM251: 4 mice, 8 slices, 175 s, 818 ns
Decreased events in s: event number × time (base, AM251, wash)	Friedman	χ^2^(2) = 83.089, *p* < 0.0001	
Base to AM251	Wilcoxon signed-rank	Z = –7.621, *p* < 0.0001	
Base to wash	Wilcoxon signed-rank	Z = –3.732, *p* < 0.0001	
AM251 to wash	Wilcoxon signed-rank	Z = –2.275, *p* = 0.001	
Decreased events in ns: event number × time (base, AM251, wash)	Friedman	X^2^(2) = 394.339, *p* < 0.0001	
Base to AM251	Wilcoxon signed-rank	Z = –16.763, *p* < 0.0001	
Base to wash	Wilcoxon signed-rank	Z = –8.882, *p* < 0.0001	
AM251 to wash	Wilcoxon signed-rank	Z = –7.578, *p* < 0.0001	
Increased events in s: event number × time (base, AM251, wash)	Friedman	χ^2^(2) = 54.926, *p* = 0.000	
Base to AM251	Wilcoxon signed-rank	Z = –6.740, *p* < 0.0001	
Base to wash	Wilcoxon signed-rank	Z = –3.090, *p* = 0.002	
AM251 to wash	Wilcoxon signed-rank	Z = –2.953, *p* = 0.003	
Increased events in ns: event number × time (base, AM251, wash)	Friedman	X^2^(2) = 302.035, *p* = 0.000	
Base to AM251	Wilcoxon signed-rank	Z = –14.338, *p* < 0.0005	
Base to wash	Wilcoxon signed-rank	Z = –5.648, *p* < 0.0005	
AM251 to wash	Wilcoxon signed-rank	Z = –9.564, *p* < 0.0005	

Both soma and non-soma regions responded similarly in the regions in which AM251 decreased the spontaneous Ca^2+^ event frequency, with the number of events decreasing during treatment and a significant recovery during 3 min of washout (Fig. 4*C*; Table 4). Similarly, soma and non-soma regions with an increased frequency of Ca^2+^ events saw a return to baseline after treatment (soma: base: 1.7 ± 0.1 events, treat: 3.5 ± 0.2 events, wash: 2.7 ± 0.2 events; non-soma: base: 2.0 ± 0.1 events, treat: 4.0 ± 0.1 events, wash: 2.7 ± 0.1 events; Table 4) indicating that AM251 blockade of endocannabinoid signaling is transient and washes out. Inhibition of the spontaneous Ca^2+^ signals demonstrates the presence of endogenous cannabinoid signaling in the SCN slice.

**Figure 5.**
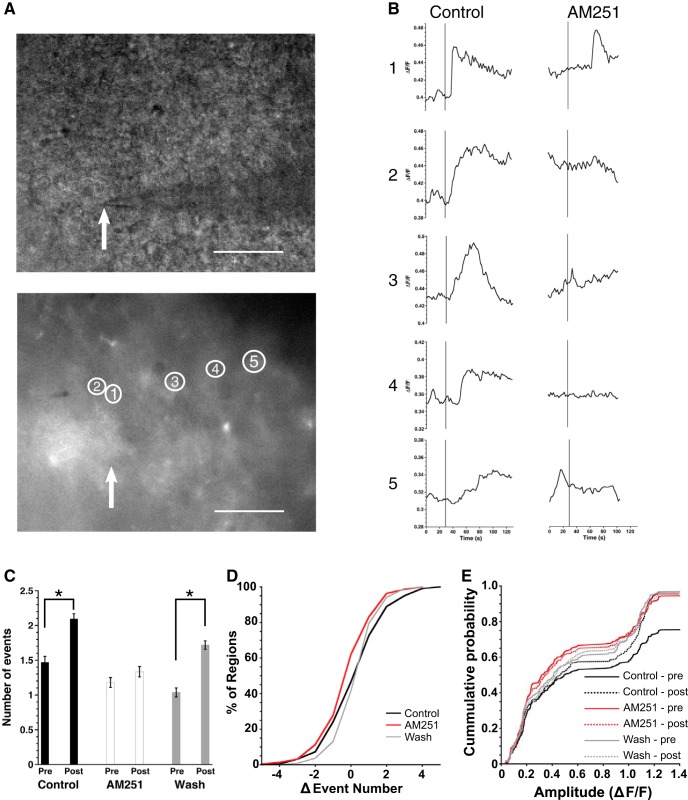
Neurons utilize endocannabinoid signaling to activate astrocyte Ca^2+^ signaling pathways. ***A***, Representative bright field (top) and GCaMP6 (bottom) images from a single slice. White scale bar is 50 μm, images taken at 40×. White arrow indicates the placement of the microelectrode tip. Numbered ellipses correspond to locations of traces in ***B***. ***B***, Representative intensity changes from the regions marked in ***A*** before and after depolarization of an SCN neuron (indicated by black bar) before (left) and during (right) AM251 (5 μM) treatment. ***C***, Average number of events pre- and post-depolarization, before, during, and after treatment with AM251 across all experiments (mean ± SEM, *p < 0.05, Friedman test). ***D***, Cumulative percentage of regions per change in event number from depolarization, before, during, and after AM251. ***E***, Boxplot of event amplitudes pre- and post-depolarization, before, during, and after treatment with AM251.

Endocannabinoid signaling acts as a retrograde signal from a postsynaptic neuron to presynaptic axon terminals, and our model suggests that in the SCN astrocytes are necessary for this process, responding to a postsynaptic neuronal release of endocannabinoids with a Ca^2+^ signaling event. To test this hypothesis, neurons were recorded in whole cell patch clamp mode and voltage-clamped at –60 mV, then depolarized (10 pulses from −80 to +20 mV, 100 ms duration, 5 Hz) while Ca^2+^ events were recorded from GCaMP6 expressing astrocytes (Fig. 5). The number of Ca^2+^ events 30 s before and after neuronal depolarization were compared before, during and after AM251 application to determine whether depolarization increased Ca^2+^ events in a CB1R-dependent manner. Before cannabinoid receptor blockade, the depolarization protocol significantly increased the number of spontaneous Ca^2+^ events (pre-depol: 1.47 ± 0.08 events, post-depol: 2.10 ± 0.07 statistics in Table 4). AM251 treatment prevented the depolarization-induced increase in Ca^2+^ event frequency (pre-depol: 1.18 ± 0.07 events, post-depol: 1.33 ± 0.07 events), an effect that recovered after 3 min of washout (pre-depol: 1.04 ± 0.06 events, post-depol: 1.72 ± 0.06 events; Fig. 5*C*,*D*). There were no differences in event amplitude distributions among any groups (Fig. 5*E*). These experiments demonstrated that depolarization of a postsynaptic neuron activated Ca^2+^ signals in astrocytes that were dependent on endocannabinoid signaling.

### Adenosine signaling is necessary for WIN response

**Figure 6.**
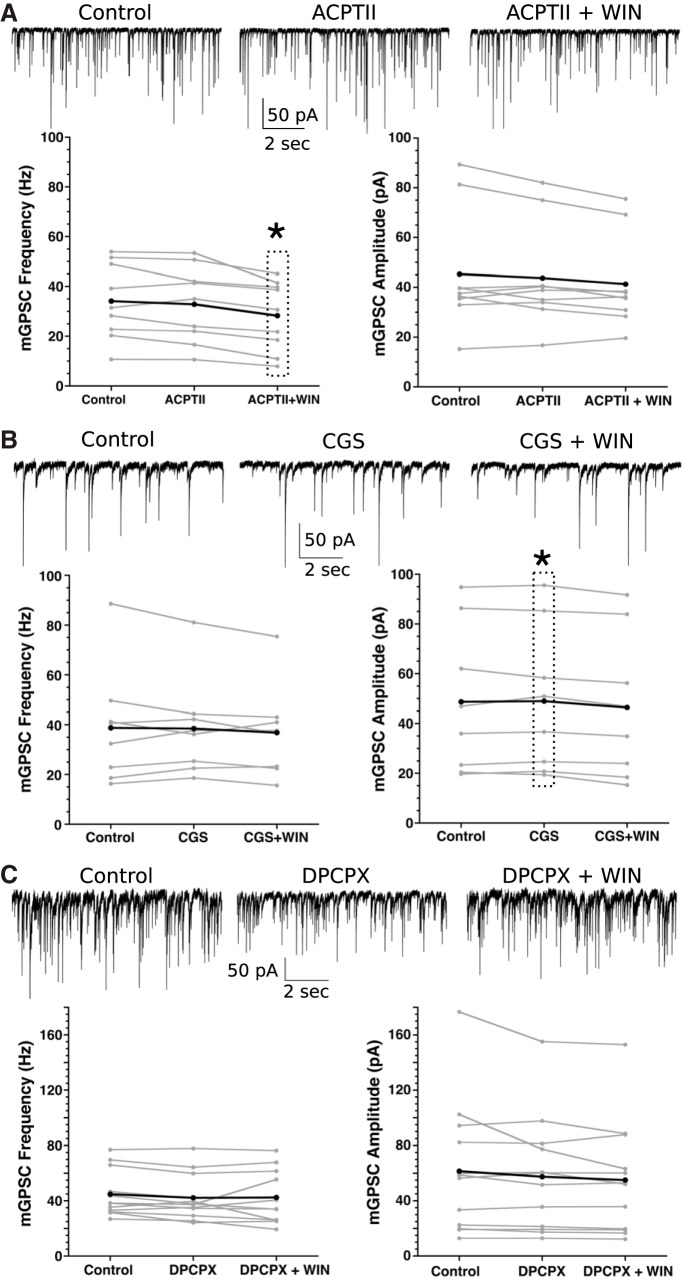
Adenosine signaling is necessary for WIN-induced mGPSC frequency changes. ***A***, top, Representative traces pretreatment (left), with ACPTII (middle), and with ACPTII+WIN (right) from an individual neuron. Bottom, Individual cell mGPSC frequency (left) and amplitude (right) before, during application of ACPTII (200 μM), and both ACPTII (200 μM) + WIN (3 μM; *p < 0.05, repeated measures analysis). ***B***, top, Representative traces pretreatment (left), with CGS15943 (CGS, middle), and with CGS +WIN (right) from an individual neuron. Bottom, Individual cell mGPSC frequency and amplitude pretreatment, with CGS (50 μM), and both CGS (50 μM) and WIN (3 μM). ***C***, top, Representative traces (left) pretreatment, with DPCPX (middle), and with DPCPX +WIN (right) from an individual neuron. Bottom, Individual cell and mean mGPSC frequency (left) and amplitude (right) pretreatment, with DPCPX (0.2 μM), and both DPCPX (0.2 μM) and WIN (3 μM). For all frequency and amplitude plots, gray lines indicate individual neuron responses, black lines represent group averages.

Cannabinoid signaling, both via endocannabinoids and the synthetic cannabinoid WIN, activated a Ca^2+^ response in astrocytes, and WIN can decrease release of GABA in SCN neurons in an astrocyte-dependent manner. Next, we wanted to identify the mechanism by which astrocytes decrease presynaptic GABA release. In other areas of the brain, astrocytes release glutamate that alters presynaptic function by activating metabotropic glutamate receptors (Liu et al., 2004; Perea and Araque, 2007; Rusakov, 2015). We demonstrated that this is not the case in the SCN by using ACPTII, a competitive metabotropic receptor inhibitor. ACPTII alone did not significantly alter mGPSC frequency or amplitude. However, on addition of WIN the mGPSC frequency significantly decreased (ACPTII+WIN: –19.2 ± 4.5%, statistics in Table 5) without significantly altering amplitude, indicating metabotropic glutamate receptors are not required for cannabinoid-induced astrocyte-mediated presynaptic changes (Fig. 6*A*; Table 5).

**Table 5.** Statistics for the data shown in Figures 5-Figures 8

Figure	Statistical test	Statistics output	*N*
Fig. 5			Depol + AM251: 9 neurons, 4 mice, 225 ROIs [soma (s) and non-soma (ns)]
Change in events: events (pre/post depol) × time (base, AM251, wash)	Friedman	χ^2^(5) = 155.838, *p* < 0.0005	
Pre to post base	Wilcoxon signed-rank	Z = –5.418, *p* < 0.0005	
Pre to post AM251	Wilcoxon signed-rank	Z = –7.401, *p* < 0.0005	
Pre to post Wash	Wilcoxon signed-rank	Z = –1.755, *p* = 0.079	
Fig. 6*A*			ACPTII: 9 neurons, 3 mice
mGPSC frequency: DMSO × ACPTII	ANOVA	*F*_(1,28)_ = 0.746, *p* = 0.395	
mGPSC amplitude: DMSO × ACPTII	ANOVA	*F*_(1,28)_ = 2.135, *p* = 0.155	
mGPSC frequency: ACPTII × ACPTII + WIN	ANOVA	*F*_(1,8)_ = 19.527, *p* = 0.002	
mGPSC amplitude: ACPTII × ACPTII + WIN	ANOVA	*F*_(1,8)_ = 3.206, *p* = 0.067	
Fig. 6*B*			CGS: 8 neurons, 3 mice
mGPSC frequency: DMSO × CGS15943 (CGS)	ANOVA	*F*_(1,28)_ = 1.539, *p* = 0.225	
mGPSC frequency: CGS × CGS + WIN	ANOVA	*F*_(1,7)_ = 1.754, *p* = 0.227	
Fig. 6*C*			DPCPX: 12 neurons, 4 mice
mGPSC frequency: DMSO × DPCPX	ANOVA	*F*_(1,31)_ = 0.03, *p* = 0.863	
mGPSC amplitude: DMSO × DPCPX	ANOVA	*F*_(1,31)_ = 0.032, *p* = 0.858	
mGPSC frequency: DPCPX × DPCPX + WIN	ANOVA	*F*_(1,11)_ = 0.007, *p* = 0.935	
mGPSC amplitude: DPCPX × DPCPX + WIN	ANOVA	*F*_(1,11)_ = 2.353, *p* = 0.153	
Fig. 7			Adenosine: 15 neurons, 4 mice
mGPSC frequency: DMSO × adenosine	ANOVA	*F*_(1,32)_ = 4.144, *p* = 0.05	
mGPSC amplitude: DMSO × adenosine	ANOVA	*F*_(1,32)_ = 4.041, *p* = 0.053	
Fig. 8			CNO: 7 slices, 54 s, 265 ns
Increase magnitude CNO: s × ns	Mann–Whitney *U*	*U* = 2667, *p* = 0.95	
Increase magnitude: treatment (CNO/ Thap + CNO) × ROI (s/ns)	Kruskal–Wallis	H(3) = 15.400, *p* = 0.001	Thap + CNO: 3 mice, 7 s, 64 ns
Increase magnitude ns: CNO × Thap + CNO	Median *post hoc*	*p* = 0.022	

Astrocytes can release ATP that can then be converted to adenosine and activate adenosine receptors (Ben Achour and Pascual, 2012; Losi et al., 2014; Yamashiro et al., 2017; Svobodova et al., 2018). CGS15943, a potent A1 and A2A adenosine receptor antagonist, was used to test the hypothesis that adenosine receptor signaling is necessary for the effects of WIN in the SCN (Fig. 6*B*; Table 5). Initially, CGS15943 (50 μM) alone did not significantly alter mGPSC frequency (Tables 1, 5), and had negligible impact on mGPSC amplitude (CGS15943: 1.3 ± 1.8%; Tables 2, 5). More interestingly, WIN had no effect on mGPSC frequency with CGS15943 in the bath (CGS15943+WIN: –0.5 ± 0.5%). Once again, the mGPSC amplitude showed very little change (CGS15943+WIN: –6.0 ± 3.0%). Next, because CGS15943 activates multiple adenosine receptors, we used the adenosine-1 receptor (A1R)-specific antagonist DPCPX to determine whether A1R, specifically, mediated the response to WIN. DPCPX (0.2 μM) alone altered neither the mGPSC frequency nor amplitude. WIN (3 μM) application in the presence of DPCPX (0.2 μM), did not alter either the frequency (DPCPX+WIN: –6.5 ± 6.1%) nor amplitude (Fig. 6*C*; Tables 2, 5), indicating that the presynaptic effects of cannabinoids are due to A1R activation.

**Figure 7.**
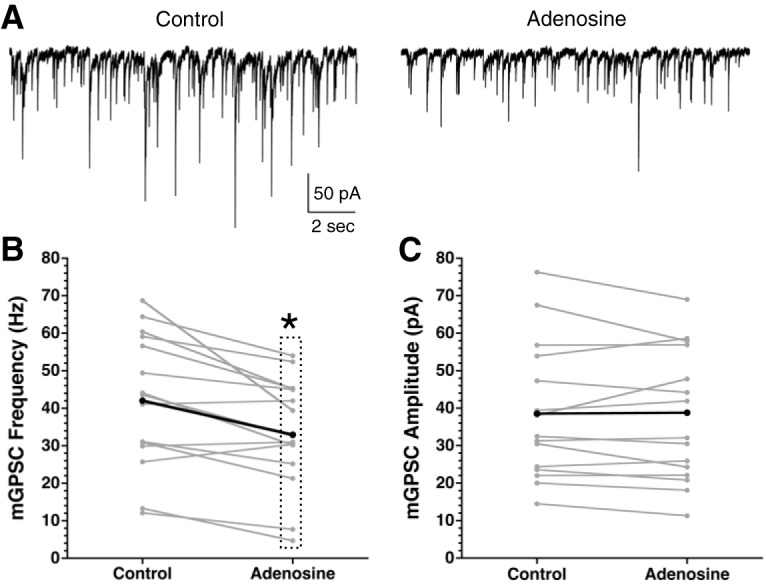
Adenosine decreases the mGPSC frequency. ***A***, Representative mGPSC recordings from a single SCN neuron before and during adenosine (100 μM) application. Frequency (***B***) and amplitude (***C***) of mGPSCs before and during adenosine treatment. Gray lines are individual cells, black represents the group average; *p = 0.05.

If the effects of cannabinoids are dependent on astrocyte function and adenosine receptor activation, then adenosine should have similar effects as WIN on mGPSC frequency. Indeed, adenosine (100 μM) causes a significant reduction of mGPSC frequency compared to controls (adenosine: –20.5 ± 5.3%) but does not significantly alter mGPSC amplitude (Fig. 7; Tables 1, 2, 5).

### Activation of a Ca^2+^ response in astrocytes changes neuronal function in the SCN

**Figure 8.**
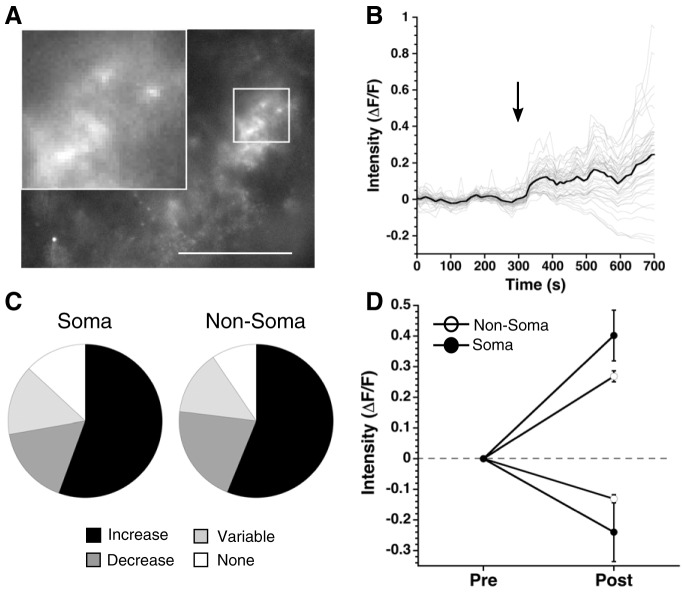
Activation of Gq DREADDs increases intracellular Ca^2+^ in astrocytes. ***A***, Representative SCN slice of a GFAP-Cre+ mouse injected with Gq DREADD and GCaMP6. Scale bar is 50 μm, images taken at 40× and the inserts magnified 120×. ***B***, Fluorescent intensity over time of the slice shown in ***A***. Black arrow indicates the CNO (10 μM) application. Gray lines are individual regions, the black line is the average response of regions. ***C***, Pie charts depict the percentage of regions that increase (black), decrease (dark gray), have a variable response (light gray), or do not respond (white) to CNO treatment across all experiments. ***D***, Average fluorescent intensity before and after CNO application displayed as mean ± SEM for soma and non-soma regions.

After demonstrating that cannabinoids increase [Ca^2+^]i in SCN astrocytes and release adenosine, we next asked whether activation of astrocytic Ca^2+^ signaling pathways could cause an adenosine-dependent decrease in mGPSC frequency independent of cannabinoid signaling. Designer receptors exclusively activated by designer drugs (DREADDs) are mutated muscarinic acetylcholine receptors that respond only to CNO, a metabolite of acetylcholine not traditionally found in mice, allowing for cell type specific expression and activation of G-protein signaling (Rogan and Roth, 2011). We expressed Gq coupled DREADDs together with GCaMP6 in GFAP-Cre+ astrocytes (Fig. 8). Application of the DREADD agonist CNO increased [Ca^2+^]i in 55.6% of soma regions (13% no response, 16.7% decrease, and 14.8% variable), and produced a similar response in non-soma regions (56.2% increase, 9.4% no response, 20.8% decrease, and 13.6% variable, statistics in Table 5; Fig. 8*C*). The magnitude of the [Ca^2+^]i increases was not significantly different between soma and non-soma regions (soma: 0.402 ± 0.0828 ΔF/F, non-soma: 0.269 ± 0.018 ΔF/F; Fig. 8*D*). After depleting Ca^2+^ stores with thapsigargin (1 μM), the magnitude of the CNO-induced Ca^2+^ responses in the soma and non-soma regions was reduced (soma: 0.126 ± 0.007 ΔF/F, non-soma: 0.169 ± 0.038 ΔF/F). Similar to the WIN + thapsigargin experiment, this effect was significant in the non-soma groups but not the soma groups due to smaller sample size of cell body regions after Ca^2+^ depletion with thapsigargin (Table 5). Application of CNO in the absence of Gq DREADD expression had no effect on the mGPSC amplitude (CNO without DREADD: −4.8 ± 1.4%) or frequency (CNO without DREADD: −10.2 ± 2.1%) compared to DMSO controls (Table 6). Because CNO did not alter the mGPSC frequency or amplitude when applied without the Gq DREADD, and DMSO does not significantly increase GCaMP6 signaling (Fig. 3) we did not investigate the effects of CNO (10 μM) on GCaMP6 signaling in the absence of DREADD expression.

**Figure 9.**
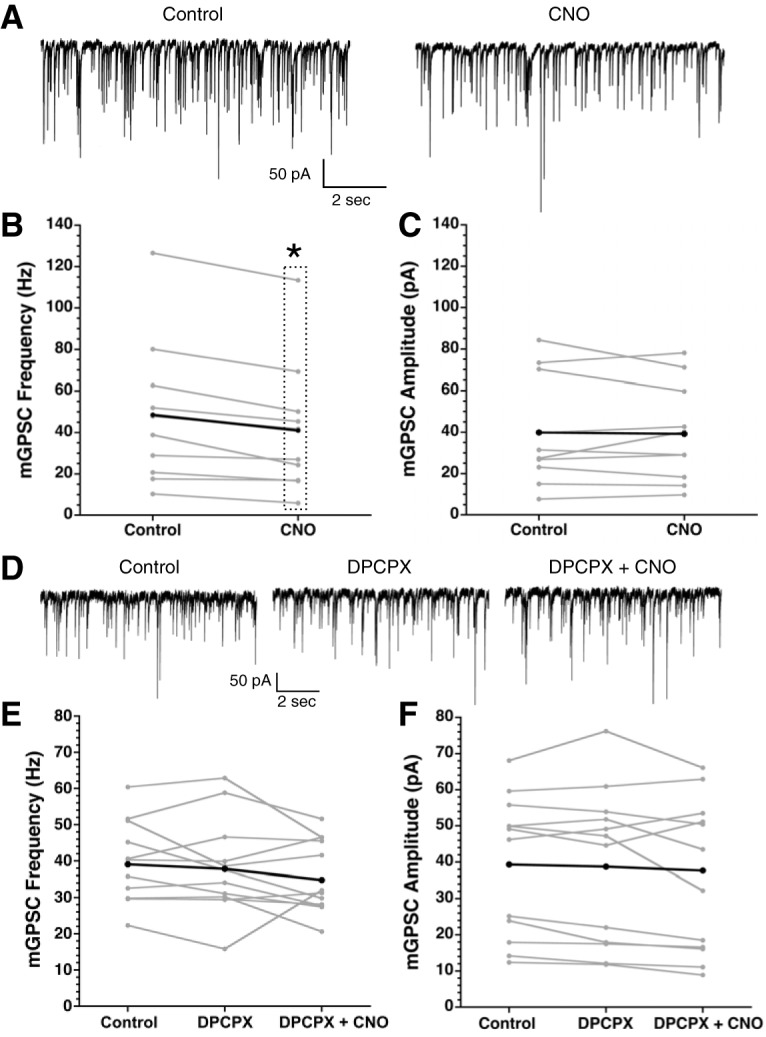
Astrocytic Gq DREADD activation decreases mGPSC frequency in an A1R-dependent manner. ***A***, Representative mGPSC traces before and after CNO (10 μM) application from a single neuron. Frequency (***B***) and amplitude (***C***) of mGPSCs before and after CNO (10 μM) treatment (*p < 0.05, repeated measures analysis). Gray lines are individual neurons, the black line is the group average. ***D***, Representative mGPSC recordings before treatment, with DPCPX (0.2 μM), and with both DPCPX (0.2 μM) + CNO (10 μM) from a single neuron. Frequency (***E***) and amplitude (***F***) of mGPSCs before treatment, with DPCPX, and DPCPX+CNO. Gray lines are individual neurons, the black line is the group average.

We hypothesized that activating a Ca^2+^ response in astrocytes would decrease presynaptic GABA release in the SCN because the Gq DREADD activation increased [Ca^2+^]i similar to that induced by WIN. Indeed, mGPSC frequency was significantly reduced in CNO treated groups compared to controls (CNO: –18 ± 4%) while the amplitude showed no difference (Fig. 9*A–C*; Tables 4-Tables 6). The reduction of mGPSC frequency by WIN was eliminated by blocking A1Rs with DPCPX (DPCPX+CNO: –6.1 ± 6.5%; Table 6) while the mGPSC amplitudes were not affected (Fig. 9*D–F*; Tables 1, 6), indicating that like cannabinoid signaling, activation of a Ca^2+^ response in astrocytes initiates a presynaptic inhibition of GABA release dependent on adenosine receptor signaling.

**Table 6.** Statistics for the data shown in Figures 9, 10

Figure	Statistical test	Statistics output	*N*
Fig. 9			
mGPSC frequency: DMSO × CNO	ANOVA	*F*_(1,30)_ = 5.642,*p* = 0.024	CNO: 10 neurons, 3 mice
mGPSC amplitude: DMSO × CNO	ANOVA	*F*_(1,30)_ = 2.205,*p* = 0.148	
mGPSC frequency: DPCPX × DPCPX + CNO	ANOVA	*F*_(1,10)_ = 0.7, *p* = 0.422	CNO + DPCPX: 12 neurons, 4 mice
mGPSC amplitude: DPCPX × DPCPX + CNO	ANOVA	*F*_(1,10)_ = 2.515,*p* = 0.144	
mGPSC frequency: DMSO × CNOwoDREADD	ANOVA	*F*_(1,30)_ = 1.334, *p* = 0.257	CNOwoDREADD: 10 neurons, 3 mice
mGPSC amplitude: DMSO × CNOwoDREADD	ANOVA	*F*_(1,30)_ = 0.731, *p* = 0.4	
Fig. 10			
Phase shift: slice × treatment (control/WIN/DPCPX/WIN+DPCPX)	Repeated measures ANOVA	*F*_(3,28)_ = 15.014, *p* < 0.001	8 mice per group
Period change WIN experiments: pre and post only	Repeated Measures ANOVA	*F*_(1,28)_ = 0.163, *p* = 0.69	8 mice per group
Period change WIN experiments: pre and post × treatment	Repeated measures ANOVA	*F*_(1,28)_ = 0.860, *p* = 0.474	8 mice per group
Phase shift: slice × treatment (adenosine/control)	Repeated measures ANOVA	*F*_(1,13.38)_ = 5.663, *p* = 0.032	7–11 mice per group
Period change adenosine: pre and post only	Repeated measures ANOVA	*F*_(1,16)_ = 10.816, *p* = 0.005	7–11 mice per group
Period change adenosine: pre and post × treatment	Repeated measures ANOVA	*F*_(1,16)_ = 0.568, *p* = 0.462	7–11 mice per group

### Both cannabinoid and adenosine signaling change circadian clock timing

**Figure 10.**
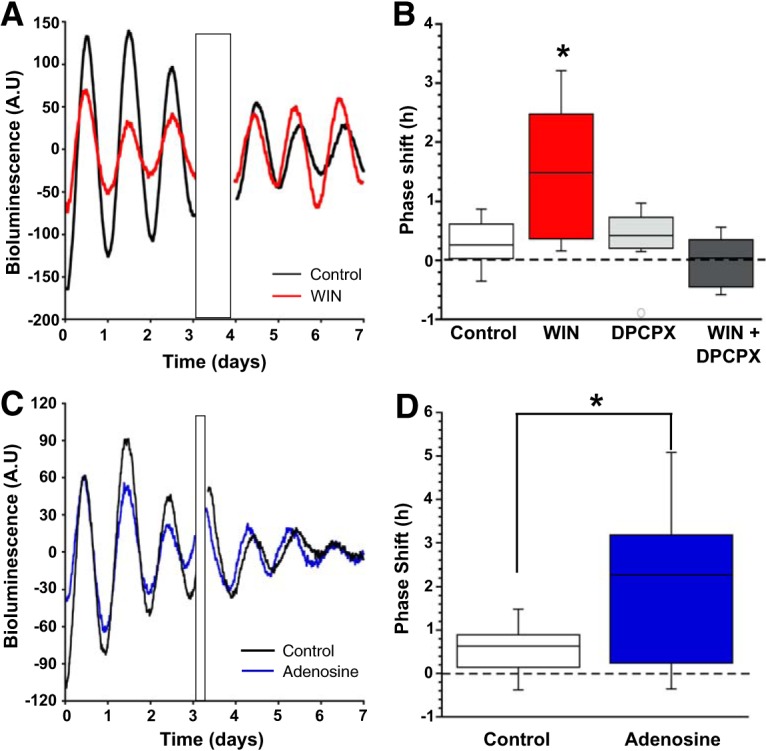
Daytime application of either WIN (3 μM) or adenosine (100 μM) phase advanced the molecular clock. ***A***, Representative bioluminescence recordings from two separate slices, one control (black) the other WIN (3 μM) treated (red), over 8 d. Treatment artifact is indicated by the black rectangle. ***B***, Box plot of phase shifts of all the cultures after treatment with either control (white) or WIN (red), DPCPX (light gray), or WIN+DPCPX (dark gray; *p < 0.05). ***C***, Representative bioluminescence recordings from two separate slices, one control (black) and the other adenosine (100 μM) treated (blue), over 7 d. Treatment artifact is indicated by the black rectangle. ***D***, Box plot of phase shifts of SCN slice cultures after treatment with either control (black) or adenosine (blue; *p < 0.05).

Cannabinoid signaling alters both astrocytic and neuronal function in the SCN. Cannabinoids also block light-induced phase shifts, but had no effect on clock timing at night (Sanford et al., 2008; Acuna-Goycolea et al., 2010). Experiments were performed to determine whether CB1R activation can phase shift the molecular clock when administered during the day using the PERIOD2::LUCIFERASE mouse model (Yoo et al., 2004). We applied WIN (3 μm) for 1 h to PER2::LUC SCN cultures during the early day (CT1–CT6). WIN significantly phase advanced PER2::LUC rhythms compared to DMSO (0.01%) vehicle-treated controls (WIN: 2.5 ± 1.3 h, vehicle: 0.2 ± 0.1 h), indicating that cannabinoids can indeed influence circadian timing (Fig. 10; Table 6). We tested the dependency of this phase shift on A1R signaling. We find the A1R antagonist DPCPX does not induce a phase shift when applied for 1 h during the day (DPCPX: 0.3 ± 0.2 h), and co-treatment of WIN and DPCPX does not produce the phase advance seen with WIN alone (DPCPX+WIN: 0.1 ± 0.2 h; Fig. 10*B*; Table 6). There was no change in period length between pre- and post-treatment under any treatment paradigm (pre-treatment period: 25.9 ± 0.1 h, post-treatment period: 25.8 ± 0.2 h; Table 6). These data indicate that cannabinoid signaling in the clock center of the brain can alter circadian timing in an A1R-dependent manner. Since the WIN-induced decrease in mGPSC frequency and phase advance of PER2::LUC rhythms are dependent on A1R activation, we hypothesized that adenosine would have similar phase shifting effects as WIN. Indeed, application of adenosine (100 μM) during the early day for 1 h significantly phase advanced the PER2::LUC rhythms compared to vehicle-treated controls (mean ± SEM, adenosine: 3.7 ± 1.7 h, vehicle: 0.5 ± 0.3 h (Fig. 10*C*,*D*; Table 6). Similar to the WIN experiments, there was no significant effect of adenosine treatment on period length (Table 6), although there was a significant shortening of period after treatment (pre-treatment period: 27.1 ± 0.4 h, post-treatment period: 25.5 ± 0.3 h) which may have been due to a different vehicle (water instead of DMSO).

## Discussion

In the SCN, postsynaptic neurons recruit astrocytes via endocannabinoid signaling to modulate presynaptic GABA release. Activation of CB1Rs with the cannabinoid agonist WIN decreases the frequency but not the amplitude of mGPSCs in the SCN, consistent with a reduction in GABA release. This effect is dependent on astrocyte function, and A1R activation. WIN also induces an astrocytic Ca^2+^ signaling cascade. Mimicking this increased Ca^2+^ signal in astrocytes using DREADD technology causes a decrease in mGPSC frequency that is also dependent on A1R activation. Finally, blockade of endogenous CB1R activation with AM251 decreases both spontaneous Ca^2+^ events, and the number of Ca^2+^ events induced by depolarization of a postsynaptic neuron indicating neuronal-derived endocannabinoid signaling modulates astrocytic Ca^2+^ signaling in the SCN. We propose a model whereby postsynaptic neuronal activity generates endocannabinoid release, activating cannabinoid receptors on astrocytes and activating an intracellular Ca^2+^ signaling pathway, causing the release of adenosine and activation of A1Rs on the presynaptic neuron to decrease GABA release. Adenosine itself decreases mGPSC frequency, supporting this hypothesis. In addition to this novel model of astrocyte recruitment to modulate GABAergic signaling in the SCN, daytime application of either WIN or adenosine phase advanced PER2::LUC rhythms, indicating a conserved mechanism for modulation of circadian timing.

There are a multitude of cannabinoids, from plant derived such as cannabidiol or (–)-*trans*-Δ^9^-tetrahydrocannabinol found in marijuana, to endocannabinoids produced by the body such as 2-arachidonoylglycerol and anandamide, to pharmacological agents designed to activate specific cannabinoid receptors (Stella, 2010; Le Boisselier et al., 2017). The affinity of these compounds for cannabinoid receptors varies greatly (McPartland et al., 2007) and cannabinoid receptors can have multiple binding sites (Lauckner et al., 2005; McPartland et al., 2007; Khajehali et al., 2015; Hua et al., 2017). To control for these confounds, we not only activated CBRs via the potent agonist WIN, but blocked CB1Rs, specifically, with AM251 to investigate the role of endocannabinoid signaling instead of relying on observations made with exogenous cannabinoids. Conventionally, CB1Rs are identified as Gi-coupled G-protein-coupled receptors, but WIN binds CB1 in such a way that it couples to Gq G-proteins and promotes release of Ca^2+^ from internal stores (Lauckner et al., 2005). Our data supports these findings such that the effects of WIN in the SCN are dependent on CB1R activation, Gq signaling increases astrocytic intracellular Ca^2+^ to a similar extent as WIN, and blockade of CB1R decreases Ca^2+^ events. Further work must be done to identify which endocannabinoid, specifically, is responsible for these Ca^2+^ events, but given the complexity of CB1R binding and function this may be difficult to discern.

Traditionally, cannabinoids act as a retrograde signal whereby after a depolarizing event a postsynaptic cell synthesizes and releases endocannabinoids that activate cannabinoid receptors on the presynaptic cell to regulate neuronal excitability (Wilson et al., 2001; Wilson and Nicoll, 2001). Our model supports previous work challenging this model, implicating astrocytes as necessary intermediates to fine tune retrograde responses (Navarrete and Araque, 2008, 2010; Min et al., 2012; Di et al., 2013; Viader et al., 2015). In the SCN, there has been growing evidence that astrocytes actively modulate neuronal function and circadian rhythmicity (Barca-Mayo et al., 2017; Brancaccio et al., 2017; Tso et al., 2017), broadening the role of astrocytes from mediating retrograde responses from neurons to actively integrating time-of-day information to the SCN network. A recent study demonstrated that astrocyte-specific deletion of BMAL1, an essential circadian gene, causes bimodal activity of animals in constant darkness. Blockade of GABA(A) over several days was able to rescue this behavioral phenotype, indicating that astrocytes regulate GABAergic signaling (Barca-Mayo et al., 2017). The current study supports these observations, demonstrating that astrocytes are necessary for modulation of GABAergic tone. Utilizing the circadian tau mutant, where a selective deletion of a mutant form of casein kinase ε in astrocytes causes a circadian period mismatch in neurons and astrocytes of 22 and 24 h, respectively, astrocytes have been shown to influence circadian free-running period, or day length (Tso et al., 2017). Future work will investigate the ability of astrocytes to synchronize SCN neurons, perhaps via cannabinoid signaling.

We chose to study cannabinoid signaling in the SCN during the day for several reasons. First, previous work demonstrated that endocannabinoids may act as a nonphotic cue (Acuna-Goycolea et al., 2010), which are more effective during the day. Next, the endogenous CB1 agonist 2-arachidonoyl-glycerol shows a diurnal rhythm, with a peak during the day (Valenti et al., 2004; Liedhegner et al., 2014). Finally, endocannabinoid signaling is different from many transmitter signaling systems in that the endocannabinoids are synthesized on demand in response to increases in neuronal activity (Navarrete and Araque, 2008), and SCN neurons are most active during the day (Green and Gillette, 1982). Astrocytes in the SCN have increased global intracellular calcium at night, with relatively little calcium signaling during the day (Brancaccio et al., 2017; Brancaccio et al., 2019). The current model suggests this increased calcium at night reflects active extracellular glutamate buffering by the astrocytes via NMDAR dependent mechanisms (Brancaccio et al., 2017, 2019). Although providing an explanation to the potential roles of astrocytes in setting periodicity and neuronal activity at night, relatively little is known about astrocytic function during the day. We suggest that decreased [Ca^2+^]_i_ during the day fits our model, enabling astrocytes to fine-tune neuronal activity locally at synapses compared to broadly buffering glutamate across the SCN. This is supported both by AM251 and FC having no direct effect on neuronal activity and by AM251 decreasing spontaneous, local astrocytic [Ca^2+^]_i_, supporting our hypothesis that endogenous cannabinoid signaling in the SCN involves astrocytes. One endogenous, physiologic condition in the SCN that could induce cannabinoid signaling may be a light stimulus that excites neurons broadly over the SCN. Future work may test the hypothesis that cannabinoid signaling in astrocytes is a mechanism to fine-tune neuronal responses to light.

## Synthesis

Reviewing Editor: Rae Silver, Columbia University Decisions are customarily a result of the Reviewing Editor and the peer reviewers coming together and discussing their recommendations until a consensus is reached. When revisions are invited, a fact-based synthesis statement explaining their decision and outlining what is needed to prepare a revision will be listed below. The following reviewer(s) agreed to reveal their identity: Mino Belle.

Work on glial cell activity in the SCN continues to reveal the pivotal roles of glia communication in circadian rhythm generation. In this study, the authors provide evidence suggesting that synaptic communication in the SCN can be regulated by endocannabinoids through glia-neuronal interactions. The mechanism may involve adenosine release from glia which acts on presynaptic adenosine-receptor type 1 to regulate synaptic transmission. For the most part, the manuscript is well and succinctly written and the experiments are well designed, and the results are clearly reported.

That said, there are some questions raised and aspects of the manuscripts that need clarification.

Importantly, both reviewers had a series of significant requests for clarification of the results and are summarized as questions about the figures. These will need substantial attention, and are listed separately for each reviewer so that the ambiguities/concerns are clear.

Materials and methods:

Page 5, Immunocytochemistry: Why was this performed in rats, while all remaining experiments were done in mice? Is this a typo error?

Results:

Page 8, line 5: Looking at table 1, I cannot decipher where the WIN and DMSO percentages are coming from. Are these correct? (-25.6{plus minus}3.6%, and -5.2{plus minus}4.3%, respectively, for example). Please check all values in the text to see if they are consistent with what is reported in the tables.

Page 11, sentence beginning with “To determine if this increase of astrocyte [Ca2+]i was......”, SCN neurons are mostly GABAergic and glutamate is also involved in SCN synaptic communication. Is there any reason why the GABA and NMDA receptor antagonists were not included in the mix to block all form of synaptic transmission?

Page 16, second paragraph, line 5: The authors stated that “The effect on mGPSC frequency was eliminated by blocking A1Rs with DPCPX....”. However, as can be seen in figure 9E, in most cells DPCPX and CNO produced highly heterogeneous responses, with some cells showing suppression while others showed elevation in mGPSC frequency. In addition, in some cells, DPCPX alone had an effect on mGPSC frequency. This would have changed the initial condition. So, such conclusion regarding the effect of CNO during the blockade of A1Rs with DPCPX is not very convincing and I believe cannot be made here, and in the discussion. Further, in some cells, DPCPX+CNO had a large effect, when compared with DPCPX alone. Thus, the effects seem to be more complex and heterogeneous than reported. Can the authors comment?

Discussion and/or abstract

With the bioluminescence experiment, the authors implied in the discussion, and stated in the abstract (line 15), that the endocannabinoid system recruits A1R signaling to phase-shift the clock and generate circadian rhythms. Although it is perfectly acceptable to predict and show that WIN and adenosine alone could have similar phase-shifting effects on clock phase, at such integrated molecular level it unfortunately does not confirm their interdependency in shaping clock phase. Other signaling pathways may be involved. To systematically exclude this possibility, it is prudent that the authors also show the effect (or lack of effect) of WIN in the presence of DPCPX on clock-phase.

Some aspects of the discussion seem somewhat far-fetched: “Perhaps endocannabinoids, which can regulate both sleep and astrocytic responses to the local neuro-environment (Metna- Laurent & Marsicano, 2015), may be critical modulators of the glymphatic system. In addition, we demonstrate daytime application of WIN 55, 212-2 and adenosine phase advances clock timing in the SCN. Astrocyte-derived circulating endocannabinoids may provide a novel mechanism to reset clock timing in areas outside of the SCN, or in whole animal behavior, providing a brain-wide time cue, influencing sleep/wake arousal states. “

It will be helpful to better position these data within the available literature as already mentioned (also see Svobodova et al 2018)

Minor comments

How did the authors ensure that there were no glial cell body signals contaminating the ROI when imaging glial processes only?

In table 4, for figure 4, should the “ns", be “s”? e.g. column 1, rows 6 and 16.

Page 13, 3rd paragraph, line 9: For clarity, the authors should state what they mean by pre and post - presumably pre step-depolarization and post step-depolarization?

Page 16, first paragraph. This should refer to table 8. Also, presumably, DREADDs expression was alongside GCAMP expression in glia? The authors should make this clear here.

In the legend of Fig. 5, the description related to Fig. 5B should be made clearer. E.g. vertical black bar. Also, indicate on the figure what the authors mean by before (left), during (middle) and after (right).

Page 7, under bioluminescence subheading, line 7: “or 0.38% sterile water using the patch clamp method”. I think there is a mistake here.

Page 4. In Electrophysiology subheading: replace “killed” with “anaesthetized or terminally anaesthetized”.

Page 4. In Electrophysiology subheading: replace “External solution” with “recording solution”?

Page 10: Could the authors comment as to why DMSO alone caused a change in astrocytic calcium levels. Is there a possibility that the DMSO concentration used was too high?

Reviewer #1 Questions raised in figures:

Fig. 1: the AM251 (CB1R antagonist) data FC data in the absence of WIN suggest that neither cannabinoid signaling (CB1R mediated, nor astrocytes are involved in SCN neuronal activation in physiological conditions, but they just may play a role in the presence of the sustained WIN-mediated pharmacological (artificial) stimulation. I believe this reduces significantly the relevance of the authors' findings. However, I note that the authors focused their recordings at ZT3-5, which is when astrocytes have low intracellular calcium and are less active (Brancaccio et al. 2017, 2019). I believe that repeating the experiments at peak astrocytic Ca2+ (CT/ZT14-CT/ZT18) may be far more informative and put these findings in a completely different light. This comment actually applies to most of the data presented in this manuscript, as the conditions are consistent throughout.

Fig. 2: It is not possible to recognize the morphology of the SCN in the figures and there is no quantification of the co-localization. Rigorous colocalization rates showing how many GFAP expressing cells also express the cl Sen and the other relevant rates) should be provided for the reader to evaluate what is claimed here. The morphology of the SCN should be appreciable in the micrographs and insets should clearly show such colocalization, or lack of there of. Moreover, if the NFHC is only localized in a part of the SCN, as the figure 2G might suggest, it may not be an appropriate neuronal marker for the colocalization analysis. If that is indeed the case the authors should comment appropriately. The author should also provide a reference to NFHC as a neuronal marker, which appear to be missing in the text.

Fig 3 and 4. The manuscript would greatly benefit from a much more focused description of experiments in fig 3 and 4.

Fig.6 On page 14, the authors rule out the relevance of glutamate in the SCN in decreasing presynaptic release of GABA, based on their data on the metabotropic glutamate receptor. The authors do not recognize that other glutamatergic pre-synaptic receptors may be at work in the SCN. In fact, this is supported by recent data, showing the involvement of NMDA receptor subsets, rather than metabotropic ones (Brancaccio et al 2017, Hastings et al 2018, Brancaccio et al. 2019). The authors may therefore want to test other forms of glutamatergic signalling, not metabotropic, before ruling glutamate out. In any case, they should explicitly refer to relevant literature and place their findings in the available context.

Fig. 9 The authors use very high CNO levels, 100X the highest concentration that does not have toxic effect in the SCN (100nM) (Brancaccio et al. 2013). This is of some concern for the interpretation of these data, especially because it would appear that there is no CNO only control (No DREADD). Also, in the light of (Gomez et al Science 2017), these are particularly relevant and needed controls.

Fig. 10 SCN slice data are of very difficult interpretation. The authors claim to observe a phase advance but judging from their (representative) traces they are in presence of a shorter period (to start with) in the WIN treated slices. Moreover, the period of Per2::Luc seems to lengthen after the WIN treatment. Similar considerations apply to the adenosine data, with the aggravation of a damping oscillation after the treatment, which contaminate any interpretation of circadian parameters. I believe the authors should not only provide better representative traces, but also a proper analysis of circadian parameters (period, amplitude, robustness, phase) and discuss their findings appropriately. Furthermore, and similarly to experiments in acute slices, the authors perform their treatment when SCN astrocytes are less active: repetition of these experiments at other circadian time may be far more informative, especially if corroborated by a PRC.

Reviewer #2 Questions raised in figures:

Fig. 1A. The control group for WIN has higher synaptic frequency than the one for DMSO. Similarly, in Fig. 1B, the control group for DMSO has higher event amplitude than the one for WIN. Can the authors comment as to why these respective control groups are different? Could these have biased the results?

Could Fig. 1F (frequency) be plotted from 0 to 80, so that the effect of FC can be seen clearer.

Fig. 2: Chloride or Cl- sensor

Figures 3, 4, 5 and 8: Please insert an inset in each image panels to show magnified glial cell bodies and processes. Please add magnification at which imaging was done in the legends too. I do appreciate that this is mentioned in the method section, but not in an obvious way.

Fig. 4C, I do not think these two panels are needed if they are from one slice.

In fig 4B, it is hard to see any changes caused by AM251. The authors should choose more representative traces.

Why is the amplitude in Fig 4F and 5E plotted differently, using cumulative probability graphs and boxplots? Please standardize data plotting for comparisons.

Fig 4. Title for the legend of Fig. 4 does not seem appropriate as the figure shows diverse responses to AM251 (E), both elevation and suppression.

The frequency of the synaptic events in some of the recordings are very high, with large event amplitude (such as those shown in Fig. 1E). Are these spontaneous release, that is, mPSCs? Were all the experiments performed in TTX, as mentioned in the materials and methods? Is there a rationale as to why the authors choose to look at mPSC rather than action potential-dependent release?

Finally, please consider providing a visual abstract, as this has been shown to increase reader attention to the paper.
